# Comparative genomics of *Clostridium bolteae* and *Clostridium clostridioforme* reveals species-specific genomic properties and numerous putative antibiotic resistance determinants

**DOI:** 10.1186/s12864-016-3152-x

**Published:** 2016-10-21

**Authors:** Pierre Dehoux, Jean Christophe Marvaud, Amr Abouelleil, Ashlee M. Earl, Thierry Lambert, Catherine Dauga

**Affiliations:** 1 Department of Genomes and Genetics, Institut Pasteur, Paris, France; 2 Faculté de Pharmacie, EA4043 “Unité Bactéries Pathogènes et Santé” (UBaPS), Université Paris Sud, Châtenay-Malabry Cedex, 92296 France; 3 Genome Sequencing and Analysis Program, Broad Institute of MIT and Harvard, Cambridge, MA USA; 4 Antibacterial Agents Unit, Department of Microbiology, Institut Pasteur, Paris, France; 5 International Group of Data Analysis, Centre for Bioinformatics, Biostatistics and Integrative Biology, Institut Pasteur, Paris, France

**Keywords:** *Clostridium bolteae*, *Clostridium clostridioforme*, Comparative genomics, Antimicrobial resistance, Butyrate pathway

## Abstract

**Background:**

*Clostridium bolteae* and *Clostridium clostridioforme*, previously included in the complex *C. clostridioforme* in the group *Clostridium* XIVa, remain difficult to distinguish by phenotypic methods. These bacteria, prevailing in the human intestinal microbiota, are opportunistic pathogens with various drug susceptibility patterns. In order to better characterize the two species and to obtain information on their antibiotic resistance genes, we analyzed the genomes of six strains of *C. bolteae* and six strains of *C. clostridioforme,* isolated from human infection.

**Results:**

The genome length of *C. bolteae* varied from 6159 to 6398 kb, and 5719 to 6059 CDSs were detected. The genomes of *C. clostridioforme* were smaller, between 5467 and 5927 kb, and contained 5231 to 5916 CDSs. The two species display different metabolic pathways. The genomes of *C. bolteae* contained lactose operons involving PTS system and complex regulation, which contribute to phenotypic differentiation from *C. clostridioforme*. The Acetyl-CoA pathway, similar to that of *Faecalibacterium prausnitzii*, a major butyrate producer in the human gut, was only found in *C. clostridioforme.* The two species have also developed diverse flagella mobility systems contributing to gut colonization. Their genomes harboured many CDSs involved in resistance to beta-lactams, glycopeptides, macrolides, chloramphenicol, lincosamides, rifampin, linezolid, bacitracin, aminoglycosides and tetracyclines. Overall antimicrobial resistance genes were similar within a species, but strain-specific resistance genes were found. We discovered a new group of genes coding for rifampin resistance in *C. bolteae. C. bolteae* 90B3 was resistant to phenicols and linezolide in producing a 23S rRNA methyltransferase. *C. clostridioforme* 90A8 contained the VanB-type Tn*1549* operon conferring vancomycin resistance. We also detected numerous genes encoding proteins related to efflux pump systems.

**Conclusion:**

Genomic comparison of *C. bolteae* and *C. clostridiofrome* revealed functional differences in butyrate pathways and in flagellar systems, which play a critical role within human microbiota. Most of the resistance genes detected in both species were previously characterized in other bacterial species. A few of them were related to antibiotics inactive against *Clostridium* spp. Some were part of mobile genetic elements suggesting that these commensals of the human microbiota act as reservoir of antimicrobial resistances.

**Electronic supplementary material:**

The online version of this article (doi:10.1186/s12864-016-3152-x) contains supplementary material, which is available to authorized users.

## Background

*Clostridium bolteae* and *Clostridium clostridioforme* are members of the normal intestinal microbiota of humans, which can cause intra-abdominal infections when the natural intestinal barrier is altered. *C. bolteae* is present in stools of most children, but counts are significantly higher in autistic children than in controls [1]. Chronic diarrheal episodes associated with some forms of autism could be attributed to an overabundance of *C. bolteae* and release of end products of metabolism, such as butyrate, propionate and acetate, that alter the motility and contraction rate of the gastrointestinal tract [2].

The two opportunistic pathogens have been isolated in intra-abdominal infections, bacteremia and in abscesses and various drug susceptibility patterns have been reported [3]. Resistance to penicillin G is common but only a few *C. clostridioforme* produce a *beta*-lactamase, as detected by the nitrocefin test [4]. Most of the strains are susceptible to ampicillin-sulbactam, piperacillin-tazobactam, imipenem, and metronidazole. Resistance to clindamycin and moxifloxacin was previously reported in the complex *C. clostridioforme* [5]. In general, *C. bolteae* appears more resistant than *C. clostridioforme*, with higher MICs of penicillin G, ampicillin-sulbactam, ticarcilllin, piperacillin and piperacillin-tazobactam and more strains producing *beta*-lactamases [6]. Resistance to aminopenicillins, lincosamides and quinolones has been reported but not documented at genetic level.

Comparative analysis of 16S rRNA gene sequences of clostridial strains place *C. clostridioforme* in the subcluster XIVa of clostridia, including several non-spore-forming cocci and mesophilic spore-forming rod shaped bacteria [7]. *C. bolteae* was previously reported as a member of the *C. clostridioforme* complex including *C. clostridioforme* (formally), *Clostridium aldenense, Clostridium citroniae* and *Clostridium hathewayi* [5, 6]. A divergence of 3 % in 16S rRNA separates *C. bolteae* from *C. clostridioforme*, but few phenotypic characters distinguish the two species, such as lactose fermentation, which is a key phenotypic test [8] and little is known about their genetics.

Commensal anaerobes of gastrointestinal tract have been proposed as reservoir for various antibiotic resistance determinants [9]. However, the sequencing effort for *Clostridium* spp., mainly concerns *C. difficile* a cause of post-antibiotic diarrhoea. Only two genomes of *C. bolteae* (strains BAA613 & WAL-14578) and three of *C. clostridioforme* (strains 2149FAA.1, WAL-7855 and CM201.1) are available in public databases.

The aim of this study was to analyse the genomes of six clinical isolates of *C. bolteae* and six clinical isolates of *C. clostridioforme* in order to identify genetic specificities between these closely related species. We focused our analysis on (CDSs coding for) functions, which could have a physiological effect within microbiota, and provided the catalogue of antimicrobial resistance genes of the two opportunistic pathogens.

## Results and discussion

### General features of genomes reveal intra and interspecies variations

A total of 1 to 21 contigs were generated from assembly of reads from Illumina (134 to 185-fold coverage) for the six strains of *C. bolteae* (Table 1). A total of 10 to 48 contigs were generated (82 to 264-fold coverage) for the six strains of *C. clostridioforme*. Total genome size varied between species and strains. The size of *C. bolteae* ranged from 6159 kb for strain 90A7 to 6480 kb for strain 90B3 with 5833 and 6059 DNA-coding sequences (CDSs), respectively, and four 16S rRNA genes. The genome size of *C. clostridioforme* was smaller, from 5467 kb for strain 90A3 to 5970 kb for strain 90A6 with 5231 to 5916 CDSs, respectively, and four 16S rRNA genes. The phylogenetic tree based on the 16S rRNA sequences showed that the *C. bolteae* and *C. clostridioforme* studied were closely related to *C. hathewayi*, *C. aldenense, C. citroniae*, *C. saccharolyticum* and *C. symbiosum*, members of the *Clostridium* cluster XIVa of Firmicutes, as previously reported [6, 7] (data not shown).

**Table 1:** Sequencing statistics and genome information

	Strain name	Year of isolation	Origin	CIP	Average sequencing coverage (times)	No. of contigs	Genome size (kb)	Predicted no. of genes (Broad Institute)	Predicted no. of proteins (Broad Institute)	No. of strain-specific CDSs (singletons & paralogs)	No. of strain-specific CDSs (unique)
*C. bolteae*	90A5	2009	perineal abcess	110254	176	12	6394	5833	5716	84	73
*C. bolteae*	90A7	2009	intraabdominal fluid	110239	185	8	6159	5719	5592	743	735
*C. bolteae*	90A9	2009	douglas abcess	110242	134	1	6367	5833	5731	163	161
*C. bolteae*	90B3	2009	pelvis abcess	110247	176	4	6480	6059	5949	239	188
*C. bolteae*	90B7	2010	pleural abcess	110255	165	19	6398	5876	5761	120	105
*C. bolteae*	90B8	2010	ureteral abcess	110256	76	21	6449	6023	5901	905	846
*C. clostridioforme*	90A1	2009	intraabdominal fluid	110245	145	16	5769	5669	5546	130	72
*C. clostridioforme*	90A3	2009	sigmoid abcess	110246	135	11	5467	5231	5096	31	21
*C. clostridioforme*	90A4	2009	bone biopsy	110238	264	48	5820	5726	5547	97	72
*C. clostridioforme*	90A6	2009	intraabdominal fluid	110240	167	22	5970	5851	5711	307	273
*C. clostridioforme*	90A8	2009	intraabdominal fluid	110249	82	47	5927	5916	5734	1190	1006
*C. clostridioforme*	90B1	2009	rectal abcess	110244	174	10	5533	5308	5184	48	36

Genomes of *C. bolteae* and *C. clostridioforme* are large genomes, where genetic redundancy is prevalent (data not shown). The redundant genes were involved in a variety of metabolic pathways, including carbon metabolism, transport, iron metabolism and amino acid biosynthesis. The differences in the number of CDSs between genomes reflected variation in genetic redundancy more than gain or loss of particular functions. In addition, genomes integrated mobile elements *i.e.* transposons, Insertion Sequences, plasmids or phages (integrase, capsid protein,…) indicative of lateral gene transfers. Some of them carried antimicrobial resistance genes (see below).

To examine the pangenome of the two species, we compared the 97,210 CDSs obtained from the 12 newly sequenced genomes with those of five other genomes (*C. bolteae* BAA613, *C. bolteae* WAL-14578, *C. clostridioforme* CM201.1*, C. clostridioforme* 2149FAA.1, and *C. clostridioforme* WAL-7855). All CDSs were clustered using the BlastClust algorithm at high stringency, above a 90 % sequence identity cut-off and 90 % length overlap. A total of 10,530 clusters were found. Only 2294 (21.78 %) clusters were shared by the two species.

In using only genomes newly sequenced, we estimated the (species) core genome and (strain-specific) genes of the six *C. bolteae*, and the six *C. clostridioforme* (Table 1). A total of 3714 genes formed the core genome of *C. bolteae*. The number of strain-specific genes in this species varied from 73 to 846. In *C. clostridioforme*, 3660 genes defined the core genome. A total of 2409 clusters were shared by the two species; 1305 genes were specific to *C. bolteae* and 1251 to *C. clostridioforme*.

*C. bolteae* 90A7 and 90B8 had the largest number of unique genes for this species (735 and 846, respectively). *C. clostridioforme* 90A8, with 1006 (17 %) unique genes had the largest number of strain specific genes in this study. These strains integrated a high number of mobile elements*.* Some unique CDSs were annotated as transporters or regulators. Few of them were involved in defence mechanisms (antimicrobial resistance genes..) or metabolic pathways. Most of them, often surrounded by CDSs from phages or transposons, were of unknown functions (data not shown).

### Functional differences between species in the core genomes

The challenge of our study was to provide reliable information from draft genomes. Therefore, we focused our analysis on the core genomes. The classification of the CDSs according to the Clusters of Orthologous Groups (COGs) system allowed to give an overview of the functions displayed by the two species. The core genomes of *C. bolteae* and *C. clostridioforme* were enriched (over 7 % of total COG matched counts) in COG categories K, E, G and R relative to Transcription (309 and 290 CDSs), Amino acid transport and metabolism (335 and 276 CDSs), Carbohydrate transport and metabolism (431 and 425 CDSs) and General function prediction (366 and 331CDSs) (Table 2)*.*

**Table 2:** Functional profile ( COG categories ) of *C. bolteae* and *C. clostridioforme*

	Core genome of *C. bolteae*	Core genome of *C. clostridioforme*	Shared between the two species	Specific of *C. bolteae*	Specific of *C. clostridioforme*
Total Number of Orthologs groups	3714	3660	2409	1305	1251
Ortholog groups in COGs	3060	2861	2151	909	710
No Hits on COGs	660	805	264	396	541
INFORMATION STORAGE AND PROCESSING					
Translation, ribosomal structure and biogenesis (J)	151	148	137	14	11
Transcription (K)	309	290	191	118	99
Replication, recombination and repair (L)	111	135	85	26	50
CELLULAR PROCESSES AND SIGNALING					
Cell cycle control, cell division, chromosome partitioning (D)	43	43	36	7	7
Defense mechanisms (V)	84	72	52	32	20
Signal transduction mechanisms (T)	180	139	101	79	38
Cell wall/membrane/envelope biogenesis (M)	122	137	97	25	40
Cell motility (Cell motility and secretion) (N)	34	18	0	34	18
Intracellular trafficking, secretion, and vesicular transport (U)	12	17	9	3	8
Posttranslational modification, protein turnover, chaperones (O)	84	83	72	12	11
METABOLISM					
Energy production and conversion (C)	194	155	120	74	35
Carbohydrate transport and metabolism (G)	431	425	296	135	129
Amino acid transport and metabolism (E)	335	276	238	97	38
Nucleotide transport and metabolism (F)	115	94	85	30	9
Coenzyme transport and metabolism (H)	99	89	72	27	17
Lipid transport and metabolism (I)	50	60	42	8	18
Inorganic ion transport and metabolism (P)	122	129	94	28	35
Secondary metabolites biosynthesis, transport and catabolism (Q)	12	0	0	12	0
POORLY CHARACTERIZED					
General function prediction only (R)	366	331	256	110	75
Function unknown (S)	187	178	150	37	28

While *C. bolteae* and *C. clostridioforme* are phenotypically related, the pattern of functions obtained through change in COG annotation differed between the two species (Table 2). 30 additional CDSs of the Nucleotide transport and metabolism (F), 97 CDSs of the Amino acid transport and metabolism (E) and 79 CDSs coding for Signal transduction mechanisms (T) categories were specific for *C. bolteae*. 40 CDSs coding for the Cell wall/membrane/envelope biogenesis (M), 50 CDSs for Replication, recombination and repair (L) and 18 CDSs for the Lipid transport and metabolism (I) categories were specific for *C. clostridioforme*. Differences between metabolic pathways in *C. clostridioforme* and *C. bolteae* seem to be large enough to support delineation of the species.

Among carbohydrate pathways, *C. bolteae* and *C. clostridioforme* harboured different systems for the assimilation of lactose, which differ in their phosphorylation states, intermediate metabolites, and bioenergetics (Additional file 1: Table S1). Genes coding for a *β*-galactosidase, which hydrolyzes lactose yielding glucose and galactose, were found in both species. An alternative lactose catabolic pathway, the lactose/cellobiose dependent phosphotransferase system (*lac/cell*-PTS) was found in almost all genomes of *C. bolteae*. The *lac/cell*-PTS operon, previously described in *C. acetobutylicum* [10], consists of genes for the 6-phospho-*β*-galactosidase, phosphoglycerate mutase, and lichenan operon transcriptional antiterminator and of two copies of genes for lactose/cellobiose family IIC, IIB and IIA components. By such a system, lactose is phosphorylated at the C-6 carbon and the internalized lactose 6-phosphate is degraded in galactose 6-phosphate and glucose by the 6-phospho-*β*-galactosidase. In addition, the gene for the 6-phospho-*β*-galactosidase and the genes for the lactose/cellobiose family components were lacking in *C. bolteae* 90A7. It is likely that this system, inducible by cellobiose or lactose and regulated by several repressors (described in other Gram positive bacteria [11, 12]) accounts for the lactose-negative phenotype in *C. bolteae* [6]. By using our annotation system, we detected a galactose operon repressor (GalR) among lacI-family regulators, in *C. clostridioforme* (all, except 90A8), but not in *C. bolteae*. Laboratory experiments are needed to determine how the transcription factors from the two species mediate preferences in the utilization of certain carbohydrates over others.

Other distinctive features between the two species were CDSs coding for secondary metabolites biosynthesis and transport and catabolism which were only found in *C. bolteae* (Table 2)*.*

**Fig. 1:**
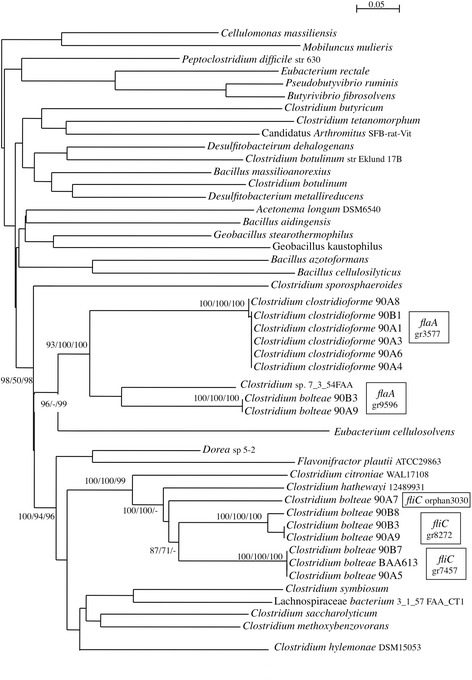
Distance-based phylogenetic tree of flagellin genes. Values at nodes corresponded to bootstrap percents obtained from phylogenetic trees based on multiple sequence alignments by ClustalW, Tcoffee or Promals, respectively. Gene names and, orphan or cluster numbers were indicated

Interestingly, the number of genes of the cell motility and secretion category (N) (34 and 18 CDSs) was different between the two species. Among them, we found CDSs encoding flagella motility recognized as essential virulence factors for most motile pathogens. Overall, twenty-four genes (46 clusters + 3 orphans) represented the flagellar operon in the genomes of *C. bolteae*. Among them, genes for flagellin (*fliC*) and flagellar cap (*fli*D), one of the multiple cell-surface adhesins of the bacteria, revealed cluster specificity and microevolution. Genes coding for fliD were represented by one cluster and two additional genes in *C. bolteae* 90A7 and 90B8. FliC sequences from *C. bolteae* 90A9, 90B3 and 90B8 formed one cluster, those from 90A5 and 90B7 clustered in another group, and sequences from *C. bolteae* 90A7 remained orphans (unique genes) after clustering (Fig. 1). They were closely related to flagellin sequences of *C. citroniae* and *C. hathewayi*, other *Clostridium* spp. of the group XIVa, isolated occasionally from human infections. In addition, *C. bolteae* 90A9 and 90B3 shared a second operon of only 19 genes in syntheny, including a flagellin gene (*fla*A) closely related to those of *C. clostridioforme* (63 % identity)*.* Based on conserved residues L87, Q88, R89 and Q96 critical for TLR5 signalling and flagellin polymerisation, these proteins were predicted to have pro-inflammatory properties [13]. In *C. clostridioforme*, twenty genes (57 other clusters) organised in a single operon encoded the flagellar apparatus*.* FlaA sequences from *C. clostridioforme* belonged to a phylogenetic group closely related to flagellin sequences from *Eubacterium cellulosovens* isolated from the rumen. Overall, flagellin genes and loci organization related to flagella were different between species (Additional file 2: Table S2 and Additional file 3: Table S3), suggesting that motility, chemotaxis, and occurrence of potential interactions with the colonic mucosa are species specific [14].

### Species differences in pathways for butyrate synthesis

Comparison of whole genome sequences revealed that pathways for butyrate synthesis, which play a key role in colonic health in humans, were present in *C. bolteae* and *C. clostridioforme*.

**Fig. 2:**
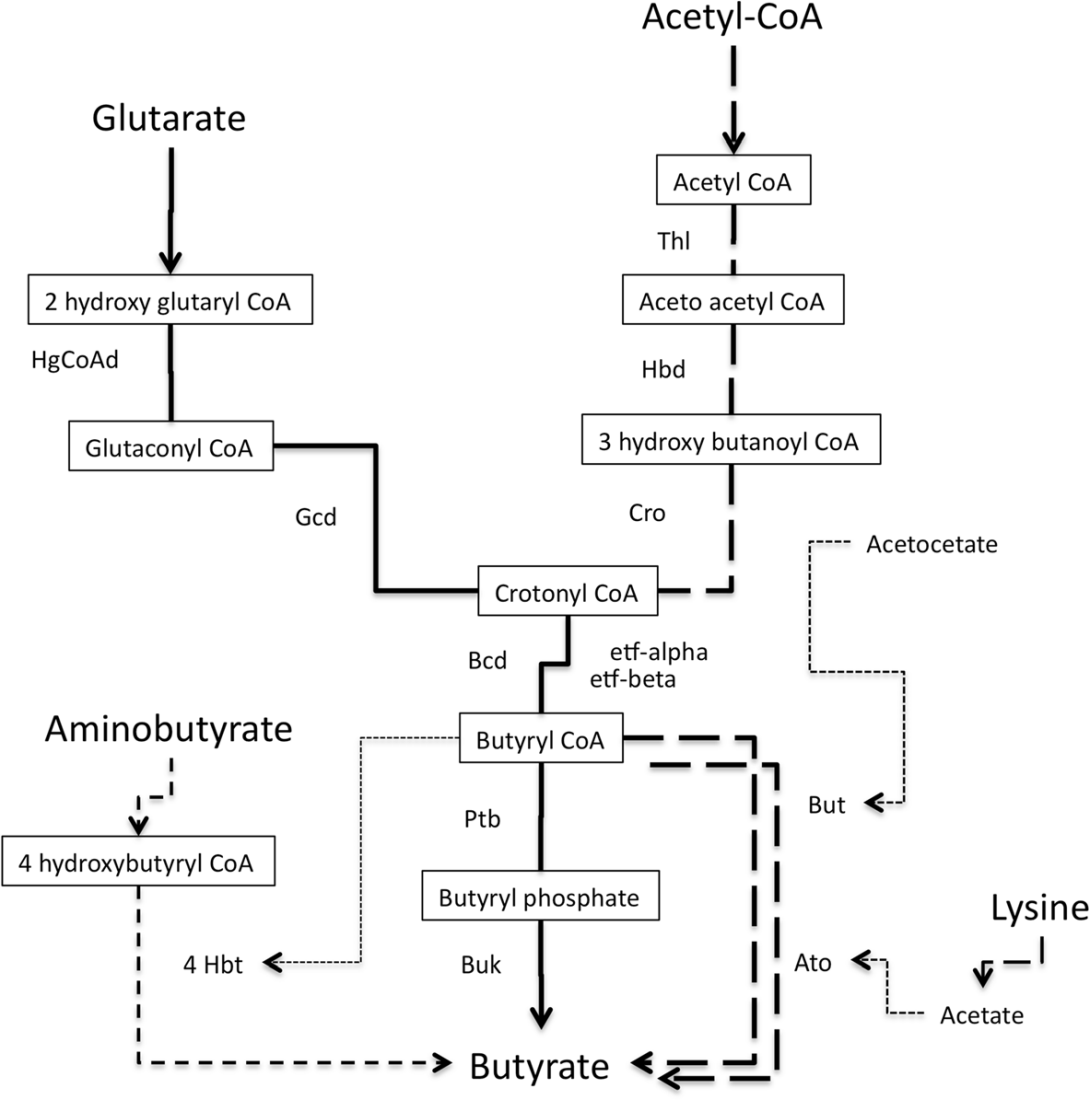
Different pathways for butyrate synthesis potentially present in genomes of *C. clostridioforme* and *C. bolteae.* In *solid lines* : found in *C. bolteae* and *C. clostridioforme*; In *bold dashed lines* : only found in *C. clostridioforme* ; In *fine dashed lines* : only found in *C. bolteae* 90A5 and 90B7 (for more details, see Additional file 4: Table S4)*.* Genes (protein names) are displayed. Ato, Acetyl-CoA acetyltransferase ; Bcd, butyryl-CoA deshydrogenase ; Buk, butyrate kinase; But, Butyrate-acetoacetate CoA-transferase ; Cro, crotonase ; Etf, electron transfer protein ; Gcd, glutaconyl-CoA decarboxylase ; Hbd, Acetoacetyl-CoA reductase ; 4Hbt, 4-hydroxybutyrate CoA transferase ; HgCoAd, 3-hydroxybutyryl-CoA dehydrogenase ; Ptb, phosphate butyryltranferase; Thl, thiolase

The two species were butyrate producers through different and complementary ways (Fig. 2, Additional file 4: Table S4). All *C. clostridioforme*, except 90A8, carried a locus coding for the Acetyl-CoA pathway (from Acetyl-CoA to butyryl-CoA), including genes for the beta-hydroxylbutyrylCoA dehydrogenase (*hbd*), thiolase (*thl*), crotonase (*cro*), butyryl-CoA dehydrogenase (*bcd*) and two electron transfer proteins (ETF *alpha*, ETF *beta*) (Additional file 4: Table S4). Only, *C. bolteae* 90A8 and *C. clostridioforme* 2149FAA.1 contained another putative *bcd* (74.9 % identity) in their genomes (data not shown). The locus composition and arrangement were similar to that in *Faecalibacterium prausnitzii*, a major butyrate producer of the human large intestine [15]. The Acetyl-CoA pathway was not found in *C. bolteae*. Both species shared genes for the two hydroxy-glutaryl-CoA dehydrogenase (HgCoAd) and the glutaconyl-CoA decarboxylase (Gcd) from the Glutarate pathway that can lead to crotonyl CoA and to butyryl-CoA *via bcd* genes [16].

The final conversion from butyryl-CoA to butyrate can be performed by the butyrate kinase (*buk*) and the phosphotransbutyrylase (*ptb*) present in both species (Additional file 4: Table S4). The group of *buk* sequences from *C. clostridioforme* branched in the vicinity of the *buk* sequence from *C. citroniae* on the phylogenetic tree (Additional file 5: Figure S1)*.* Buk sequences from *C. bolteae* formed distinct monophyletic groups and sequences distributed among the phylogenetic trees, suggesting polymorphism and/or functional variations of the enzyme in this species. Other genes for transferases from the lysine pathway (*ato—alpha* and *beta* subunit ; *but*—acetate CoA transferase), detected near the butyrate locus in six genomes of *C. clostridioforme*, can be involved as final enzymes. Genes from the 4-aminobutyrate pathway (4*hbt*) can be another alternative for the terminal step in *C. bolteae* 90A5, 90B7, WAL14578, and BAA613 [16].

The butyryl-CoA:acetate CoA-transferase (*but*) of the acetyl-CoA pathway, the final step in butyrate production predominant in *Clostridium* XIVa, was neither found in the common core genomes nor in the genomes of the *C. bolteae* studied [17]. In the human gut, previous studies on colonic isolates of healthy individuals have illustrated that *but* pathway predominates [18]. Further studies are needed to assess the impact of butyrate production through the Glutarate pathway on health of colonic cells, in particular in autism where *C. bolteae* is overabundant [2].

### Identification of antibiotic resistance determinants

Drug resistance genes that had not been recognized by automated annotation were identified by homology sequence research on ARDB. Resistance genes were predicted on a value up to 40 % identity (50 % of positive substitutions) on 70 % of length above the cut-off value usually recommended (see list in Table 3). Then, the gene content and genetic organization of microbial resistance loci of the six *C. bolteae* and the six *C. clostridioforme* were compared to previous data obtained from *C. clostridioforme* CM201.1 in our laboratory.

**Table 3:** Distribution of CDSs annotated as antimicrobial resistance genes

	ARDB	Number of clusters and orphans	*C. bolteae* 90A5	*C. bolteae* 90A7	*C. bolteae* 90A9	*C. bolteae* 90B3	*C. bolteae* 90B7	*C. bolteae* 90B8	*C. clostridioforme* 90A1	*C. clostridioforme* 90A3	*C. clostridioforme* 90A4	*C. clostridioforme* 90A6	*C. clostridioforme* 90A8	*C. clostridioforme* 90B1
class A beta-lactamase	blaCLO1	2	1	1	1	1	1	1	1	1	1	1	1	1
class B beta-lactamase	metallo-enzymes	4	1	2	1	1	1	1	2	1	2	2	2	1
class C beta-lactamase	Amp C and Penicillin Binding Protein	1	1	1	1	1	1	1	1	1	1	1	1	1
class D beta-lactamase	Amp D (oxa type)	2	1	1	1	1	1	1	1	1	1	1	2	1
Teicoplanin, Vancomycin	VanR_A_	4		2					1	1	1	1	3	1
	VanS_A_	1							1	1	1	1	1	1
	VanD	2							2	2	2	2		2
	VanS_D_	1							1	1	1	1		1
	VanX_D_	1							1	1	1	1		1
	VanR_D_	2							1	1	1	1		1
Vancomycin	VanH_B_	2	1	1	1	1	1	1	1	1	1	1	2	1
	VanR_B_	2	1	1	1	1	1	1	1	1	1	1	2	1
	VanB	1											1	
	VanS_B_	1											1	
	VanW_B_	1											1	
	VanX_B_	1											1	
	VanY_B_	2											2	
	VanS_C_	1											1	
	VanR_E_	1		1	1	1								
	VanG	1	1	1	1	1	1	1	1	1	1	1	1	1
	VanR_G_	3	2	2	2	2	2	2	1	1	1	1	1	1
	VanY_G_	1	1	1	1	1	1	1						
	VanU_G_	3	2				1							
Teicoplanin	VanZ	4	2	3	1	1	2	1	2	2	2	2	2	2
Lincomycin	LnuA	3	1	1	1	1	1	1	1	1	1	2	1	2
Macrolides	ErmB	1									1		1	
Macrolide-Lincosamide- Streptogramin (efflux pumps)	MacB	1	6	8	7	7	6	6	6	6	6	6	5	6
	MefA	1	3	2	4	4	3	3	3	3	3	4	3	3
	MsrA/MsrB	5	3		3	3	3	3						
	Lsa	1		1								1		1
ABC transporter (efflux pumps)	VgaA	1	1	1	1	1	1	1	1	1	1	1	1	1
	Tirc	1	1	1	1	1	1	1	1	1	1	1	1	1
	CcmA	6		2					2	3	3	2	4	3
Streptogramin_A Chloramphenicol	VatB	2	1	1	1	1	1	1	1	1	1	1	1	1
chloramphenicol	CAT	3	1				1	2		1	1	1	1	1
linezolide	Cfr	2	1	1		2	1		1	1	1	1	1	1
Rifampicin	Arr	1	1	1	1	1	1	1						
Metronidazole	Nim	1	1	1	1	1	1	1	1	1	1	1	1	1
Trimethoprim	Dfra20	1	1	1	1	1	1	1	1	1	1	1	1	1
	Dfra	1										1		
Aminoglycosides	Aac6ie	2												2
	Aad9ib	1				1								
	Ant6ia	3		1					1	2	1	2		1
	Aph3iiia	1								2		1		1
	Aph^a^	3	3	3	3	3	3	3	3	3	3	3	3	3
Tetracycline	Tet40	1				1						1		
	TetO	1	1	1	1	1	1	1					1	
	TetW	1							1	1		1		1
	Tet32	1									1	1		
Bacitracin	BacA	3	1	1	2	2	1	1	1	2	2	1	1	2
	BcrA	1	2	3	4	4	2	3	3	4	4	3	4	5
Multidrug resistance efflux pump	Blt	1		1										
	AcrB	3	2	2	2	2	2	2	3	3	3	3	3	3

^a^Broad annotation

Because sequence-based predictions might potentially identify determinants that do not lead to antimicrobial resistance, susceptibility testing was performed to obtain information on the predicted response of bacteria to antibiotics. The strains included in this study showed resistance patterns, including ampicillin, macrolides, lincomycin and quinolones, now common in anaerobes (Table 4). Both genomic data (CDSs and annotations) and phenotypic susceptibility tests were considered to identify antibiotic resistance determinants (Tables 3 and 4). Preliminary assays for cloning certain genes were also performed in order to check their capacity to confer antibiotic resistance (see below).

**Table 4:** Antibiogram of *C. clostridioforme* and *C. bolteae*

	*C. bolteae*	*C. bolteae*	*C. bolteae*	*C. bolteae*	*C. bolteae*	*C. bolteae*	*C. clostridioforme*	*C. clostridioforme*	*C. clostridioforme*	*C. clostridioforme*	*C. clostridioforme*	*C. clostridioforme*
Antimicrobial agent	90A5	90A7	90A9	90B3	90B7	90B8	90A1	90A3	90A4	90A6	90A8	90B1
Beta - lactams												
ampicillin	R	R	R	R	R	R	R	R	R	R	R	R
amox-clavulanic acid	S	S	S	S	S	S	S	S	S	S	S	S
cefoxitin	S	S	S	S	S	S	S	R	S	I	S	I
imipenem	S	S	S	S	S	S	S	S	S	S	S	S
Glycopeptides												
Vancomycin	S	S	S	S	S	S	S	S	S	S	R^a^	S
Macrolides												
Erythromycin	R	R	I	R	R	R	R	R	R	R	R	R
Telithromycin (Ketolides)	R	R	S	R	R	R	R	R	R	R	R	R
Spiramycin	S	S	S	R	S	I	S	S	R	R	R	S
Lincosamides												
Lincomycin	I	R	R	R	R	R	S	I	R	R	R	R
Clindamycin	S	S	S	R	S	S	S	S	R	S	R	S
Streptogramins												
Pristinamycin	S	S	S	nd	I	S	S	S	S	S	S	S
Amphenicols												
Chloramphenicol	nd	S	S	R	S	R	S	S	S	S	S	S
Oxazolidinones												
Linezolid	S	S	S	R^b^	S	S	S	S	S	S	S	S
Rifampin	S	R^c^	S	S	S	S	S	S	S	S	S	S
Quinolones												
Ciprofloxacin	R	R	R	R	R	R	R	R	R	R	R	R
Moxifloxacin	R	R	R	R	R	R	R	R	R	R	R	R
Tetracyclines												
Doxycycline	S	R	R	R	R	S	R	R	R	R	R	R
Nitroimidazole												
Metronidazole	S	S	S	S	S	S	S	S	S	S	S	S
Polypeptides												
Colistin	R	R	R	R	R	R	R	R	R	R	R	R

^a^MIC vancomycin > 250 mg/l

^b^MIC linezolid =16 mg/l

^c^MIC rifampin =32 mg/l

### Genes of resistance to antibiotics used for treatment of anaerobic infections

A total of 76 clusters and 21 strain-specific genes potentially involved in antimicrobial resistance were identified (Table 3). It’s included from 42 to 50 CDSs in *C. bolteae* and 48 to 58 CDSs in *C. clostridioforme*. From 27 to 42 CDSs per genomes were related to drug resistance mechanisms to *beta*-lactams, glycopeptides, macrolides, lincosamides, and metronidazole.

**Fig. 3:**
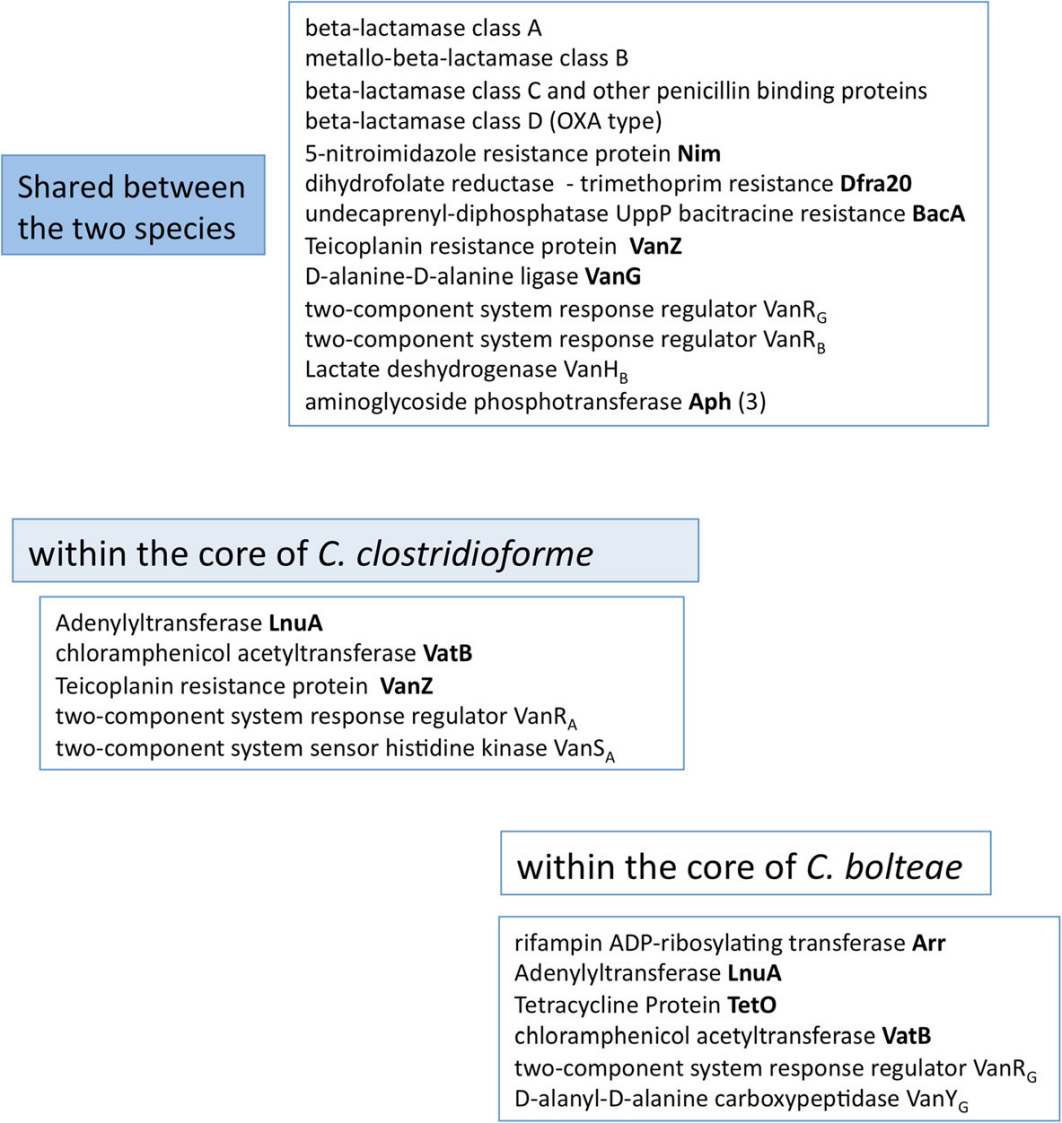
Distribution of antibiotic resistance genes shared between and within the core of *C. bolteae* and *C. clostridioforme*. Genes overlapping at least 90 % length and 90 % of similarity were considered homologs. Resistance genes were predicted on a value up to 40 % identity (50 % of positive substitutions) on 70 % of length by homology sequence research on ARDB. For all *C. clostridioforme* and some *C. bolteae* 23S rRNA methyltransferase Cfr-like

Seven clusters involved in *beta*-lactam resistance are shared or part of the core genome of the two species (Fig. 3). Three types of *beta*-lactamases, including class A *beta*-lactamase, class C *beta*-lactamase, class D [Oxa type] *beta*-lactamase and several metallo-enzymes were recognized in the twelve genomes. All the strains studied, selected for their resistance to ampicillin, shared the gene *bla*CLO1, previously found in *C. clostridioforme* CM201.1 (unpublished), but the structure of integrative conjugative element (ICE) observed in CM201.1 was not found in the new genomes sequenced. The gene *bla*CLO1 confers resistance to aminopenicillins and carboxypenicillins in *E. coli,* and its activity is inhibited by clavulanate and sulbactam. Nine amino acid changes were observed in *beta*-lactamases of *C. bolteae* 90A9, 90B3 and 90A8. This closely related *beta*-lactamase was flanked by insertion sequences (IS66) and a putative gene for class D *beta*-lactamase (COG 2602) also described in *Clostridium* sp M62/1 from the human intestinal microflora (HMP project). Genes for class C *beta*-lactamases, previously found in the chromosomes of enteric bacteria (COG2680), were also present in *C. bolteae* and *C. clostridioforme*.

A high number of predicted genes (32 CDSs) were involved in resistance to glycopeptides (Additional file 6: Figure S2). *C. clostridioforme* 90A8 was the single strain with all genes required for glycopeptide resistance in agreement with the phenotype (MIC > 256 mg/l). Vancomycin resistance in this strain was attributed to a VanB-type operon borne by a Tn*1549-like* element. Unfortunately, a deletion of ten nucleotides within the relaxase gene of Tn*1549* leads to the inability of 90A8 to transfer vancomycin resistance in vitro [19]. The other genomes of *C. clostridioforme* included part of the VanD-type vancomycin resistance operon, but the D-Ala-Lac ligase *van*D gene was disrupted by a stop codon leading to a truncated protein (Additional file 6: Figure S2). In addition *van*H and *van*Y*,* which encode a D-lactate dehydrogenase and a DD carboxypeptidase, respectively, were missing. Similarly, the genomes of *C. bolteae* harboured four CDSs, homolog of *van*R_G_ *van*U_G_ *van*G *van*Y_G_, which formed an incomplete and non-functional operon due to the lack of a serine racemase gene. The high number of CDSs coding for glycopeptide resistance (including *vanD* or *vanG* known to be chromosomal and not transferable) found in the genomes of *C. bolteae* and *C. clostridioforme*, suggests that they are part of ancestral entire operons, which have evolved in the absence of antibiotic selective pressure [20]. Presence of incomplete *van* operon is intriguing, but similar observations in other anaerobes living in microbiomes such as *Clostridium difficile* 630 or *Ruminococcus* spp., have been reported [21, 22].

Homologues to the adenylyltransferase [*lnu*A] gene conferring resistance to the lincosamides, were present in the core genome of both species (Fig. 3)*.* LnuA genes of *C. clostridioforme* and *C. bolteae* had 70 to 72 % identity with orthologs found in *C. hathewayi* and *C. citroniae*, respectively. *C. clostridioforme* 90B1 and 90A6 harboured an additional *lnu* gene (68 % identity with LnuA_90A5_), with no traces of mobile elements. Lincomycin resistance is common in *C. bolteae* and *C. clostridioforme*, often associated with resistance to clindamycin*.* LnuA proteins of the two species displayed 51 to 54 % of identity with LnuA from *Staphylococcus* suggesting common functionality*,* but the role of *lnu* genes is difficult to establish due to the presence of other putative mechanisms [23]. Similarly, all strains were resistant to erythromycin, whereas two genes homolog to the erythromycin ribosome methylase gene, *erm*B, were only predicted in the genomes of *C. clostridioforme* 90A4 and 90A8. Over-expression of multidrug efflux pumps and Macrolide-, and various Macrolide-Lincosamide-Streptogramin B—specific efflux systems, such as MacB, MefA, VgaA, MsrA/MsrB*,* and CcmA (Table 3, Additional file 7: Figure S3), found in the genomes studied, can lead to macrolide and lincosamide resistance [24]. In addition, two clusters of CDSs coding for xenobiotic acetyltransferases related to VatB (48 % identity) were found in the core genomes of each of the species (Fig. 3)*.* VatB inactivates virginiamycin [25], but here, the resistance was not detected by anti-microbial susceptibility tests (Table 4).

A cluster of CDSs homologues to the metronidazole resistance (*nim*) genes was detected in the core genomes of both species, and metronidazole was very active on the species studied [26]. In *Bacteroides fragilis* it has been demonstrated that increased expression of *nim* genes when downstream from IS elements leads to metronidazole resistance [27]. In the lack of IS directly upstream the *nim* genes, the mechanism to confer metronidazole resistance to *C. bolteae* and *C. clostridioforme* remains to be established.

### Unexpected observation of new genes of resistance

Regarding other drug resistances, genome data revealed genes related to chloramphenicol and rifampin resistance mechanisms (Table 3). In most genomes of each species, we found CDSs coding for a group A chloramphenicol acetyltransferase [*cat*] which can inactivate chloramphenicol. However, only *C. bolteae* 90B3 and 90B8 were resistant to chloramphenicol. The genome of strain 90B8 contained a second copy of the *cat* gene borne by a Tn*4451-*like transposon (96 and 90 % identity with Tn*4451* and Tn*4453*, respectively). The 90B3 genome contained a CDS homolog to the gene *cfr* coding for a 23S rRNA methyl-transferase largely spread in Gram-positive bacteria. As expected, the strain 90B3 was also resistant to florfenicol, tiamulin, and linezolid (MIC = 16 mg/l). Other 23S rRNA methyltransferase (Cfr-*like*) CDSs were detected in an environment rich in transposable elements (Tn 6103-6110-CTn4 [fragments]) in the genomes of *C. clostridioforme* and *C. bolteae* 90A5 and 90B7, but the rRNA methylation did not appear to affect the susceptibility to chloramphenicol (Fig. 3).

The analysis of genomic data allowed to recognize CDSs homologues to rifampin-ADP-ribosyltransferase (*arr*) genes in *C. bolteae* but not in *C. clostridioforme.* No mobile elements or traces of mobile elements were found around the *arr* genes suggesting they were indigenous to this species. This new Arr sequences branched in the vicinity of Arr proteins from *C. saccharoperbutylacetonicum* and some Cyanobacteria on the phylogenetic tree (Additional file 8: Figure S4). They were distinct from Arr-2 proteins of Enterobacteriaceae and from Arr proteins of *Mycobacterium* and *Streptomyces* spp.. All strains, except 90A7, were susceptible to rifampin. In the absence of mutations in *rpo*B (known to be responsible for rifampin resistance), resistance of *C. bolteae* 90A7 (MIC: 32 mg/l) was likely due to positive selection of mutations in *aar*_Cbol90A7_. Susceptibility to rifampin of other *C. bolteae* was likely due to the lack of promoters upstream from *arr* (as predicted by *in silico* analysis) or to nucleotide substitutions within *arr* leading to amino acid replacement and functional inactivation (data not shown).

Concerning the resistance of all strains against moxifloxacin and ciprofloxacin, all strains of *C. bolteae* showed several substitutions in the “quinolone-resistance–determining region” (QRDR) of *gyr*B*.* We didn’t find any substitutions in this region for *gyr*A, nor described in the protein of the quinolone-resistant epidemic strain, *C. difficile* 027 [28]. Therefore, GyrB was likely the preferred target in acquisition of quinolone resistance in these two species. In addition, several CDSs coding for AcrB inner membrane transporter were present in all the strains. These transporters are part of a resistance-nodulation-division [RND] multidrug efflux pump, known to increase efflux of quinolones in some Gram-negative bacteria [29]. Further studies are needed to determine their influence in the loss of susceptibility of *Clostridium* spp. to fluoroquinolones*.*

Overall, similar resistance profiles against antibiotics in *C. bolteae* and *C. clostridioforme* can result from various mechanisms.

### Genes of resistance to antibiotics less active or inactive against *Clostridium* spp

The genomes of *C. bolteae* and *C.clostridioforme* carried one or two copies of the undecaprenyl pyrophosphate phosphatase gene *bac*A, and 2 to 5 copies of the efflux pump genes, *bcr*A, involved in bacitracin resistance, in agreement with their low susceptibility [26]. We also found one cluster of CDSs homolog to the dihydrofolate reductase gene, *dfr*A20 (41 % identity / 97 % length) of *Pasteurella multocida* in the core genome of both species that could explain the poor activity of trimethoprim on our *Clostridium* spp. [30]. In addition, *C. clostridioforme* 90A6 harbored a CDS identical to *dfr*A from *Enterococcus faecium*. This gene detected in an environment rich in mobile elements is consistent with a new example of horizontal transfer between *Enterococcus* spp. and Clostridiales.

Interestingly, the genomes of *C. bolteae* or *C. clostridioforme* contained various resistance genes against antibiotics naturally inactive on these species. Five CDSs were homologs of genes that phosphorylate, acetylate or adenylylate aminoglycosides. Four of these putative resistance genes were detected in an environment of mobile elements. Three genes, *aadE, sat4* and *aph(3’)-III*, conferring resistance to streptothricin, streptomycin and kanamycin, respectively, was found part of a transposon delineated by two IS1182 copies in *C. clostridioforme* 90A3 (two copies), 90A6 and 90B1. The *aph(3’)-III* detected was identical to the *aph(3’)-III*, part of the multidrug resistant plasmid PF856 from *E. faecium* [31], also related to an internal domain (99 % identity) of a SSCmec element of *Staphylococcus aureus* HT20040085. Similarly, the aminoglycoside 6-adenylyltransferase gene *ant(6’)-Ia*, conferring resistance to streptomycin was shared by *C. clostridioforme* 90A1, 90A3 (2 copies), 90A4, 90A6 (2 copies) and *C. bolteae* 90A7. The adenylyltransferase gene *aad(9’)-b* which mediates resistance to streptomycin/spectinomycin was found in *C. bolteae* 90B3. Two copies of the acetyl transferase *aac(6’)-Im* were also present in the genome of *C. clostridioforme* 90B1. Homologs of AAC(6’)-Im which confers resistance to tobramycin and amikacin resistance was also found in *E. coli* (96 % identity), *Coprococcus sp, C. difficile* and *Enterococcus faecium* (data not shown). In addition, three CDSs coding for an aminoglycoside kinase (APH), known to be widely distributed in Gram-positive bacteria, were also observed among all the strains [32].

Numerous tetracycline resistance genes were also detected in *C. bolteae* and *C. clostridioforme*. They include both efflux genes such as *tet40*, and ribosome protection determinants such as *tetO*, *tetW*, and *tet32*, previously reported as circulating in gut microflora among distantly related bacteria [33].

## Conclusion

We studied the genomes of *C. bolteae* and *C. clostridioforme,* two species of the complex Clostridioforme, which can behave either as members of the human microbiota or as opportunistic pathogens*.*

We compared the genomes of six clinical isolates of *C. bolteae* and 6 strains of *C. clostridioforme* with available genome sequences in laboratory and international databanks. These data were used as a basis to reveal differences in functional patterns between the two species. Among them, differences in flagella coding genes and butyrate pathways can potentially influence host-gut microbiota interactions.

The patterns of resistance genes in the genomes were also of peculiar interest: (i) these bacterial species harbour specific and indigenous putative resistance genes, which included ABC transporters, antibiotic modifying enzymes, rRNA methyltransferases, (ii) Other resistance genes were acquired as shown by their location within mobile elements in the genomes studied. Some of them corresponded to genes mainly spread in gut bacteria. Others were new determinants, which remain to be analysed for their ability to confer antibiotic resistance. This study emphasizes the role of commensal bacteria of the digestive microbiota as reservoir for antibiotic resistances.

### Future directions

As the costs of whole-genome sequencing continue to decline, it becomes increasingly available in routine diagnostic laboratories to detect antimicrobial resistance genes in genomes as substitute of traditional methods for resistance identification. However, the true challenge will remain to extract the relevant information from the large amount of data and to ensure the functionality of the genes detected.

## Methods

### Bacterial strains, molecular Identification and antimicrobial susceptibility testing

We studied retrospectively 6 strains of *C. bolteae* and 6 strains of *C. clostridioforme*, resistant to beta-lactams. Strains were isolated from intra-abdominal infections from patients (without links) over 2 years in two hospitals, Paris Saint Joseph and CHRU of Nancy, France.

*Clostridium* isolates were grown in Brain Heart Infusion agar or broth (Oxoid) in an anaerobic atmosphere (5 % CO_2_, 5 % H_2_ and 90 % N_2_) at 37 °C. Antibiotic susceptibility was tested by disk diffusion on Mueller-Hinton (MH) medium supplemented with 5 % defibrillated horse blood, according to the standards of the Comité de l’Antibiogramme de la Société Française de Microbiologie (http://www.sfm-microbiologie.org/). MICs of antimicrobial agents were determined on MH agar by Etest (bioMérieux).

Genomic DNA for sequencing was prepared from cells by lysis in lysozyme, incubation in proteinase K/SDS, followed by a standard phenol/chloroform extraction procedure. Prior to library preparation DNA quality was assessed by Nanodrop analysis (Thermo Scientific).

Molecular identification of strains was performed by sequencing a 1483-bp PCR fragment from 16S rRNA using universal primers B27F (5’-AGAGTTTGATCCTGGCTCAG) and U1492R (5’-GGTTACCTTGTTACGACTT) [34]. The taxonomic assignment of sequences was checked on the RDP classifier of the Ribosomal Database Project v11.1 [35]. Closely related 16S rRNA sequences found in the database were aligned with the newly determined sequences and a phylogenetic tree was re-constructed according to the neighbor-joining method of the Phylip package [36]. The stability of the groupings was estimated by bootstrap analysis (100 replications). The identification of *C. bolteae* 90A7, previously misnamed *C. clostridioforme* 90A7, was corrected (see Results and discussion).

### Whole genome sequencing

This sequencing project was part of the Human Microbiome U54 initiative of the Broad Institute (broadinstitute.org). For each genome, paired end libraries were generated using Illumina’s Phusion-based library kits following the manufacturer’s protocols (Illumina, Hayward, CA, USA). Samples were multiplexed and sequenced on Illumina GAIIx machines and base-called following the manufacturer’s protocols. Individual samples of paired 90 nt reads generated 76 to 185-fold coverage of the genomes of *C. bolteae* (~6.37 Mb) and 82 to 264-fold coverage of the genomes of *C. clostridioforme* (~5.75 Mb), respectively (Table 1). Sequences were *de novo* assembled, yielding 1 to 21 contigs for genomes of *C. bolteae* and 10 to 48 contigs for those of *C. clostridioforme*.

### Accession to our set of genomic sequences of Clostridium spp in public databases

All the genomes sequenced are available on the Broad Institute website (http://www.broadinstitute.org/data-software-and-tools) (Genbank Bioprojects : PRJNA64845, PRJNA64847, PRJNA64849, PRJNA64851, PRJNA64853, PRJNA64857, PRJNA659, PRJNA64861, PRJNA64863, PRJNA64865, PRJNA64867, PRJNA64869). The genomes of *C. beijerinckii* NCIMB 8052 (*Clostridium sensu stricto), C. saccharolyticum* WM1, *C. bolteae* BAA613 were retrieved from the PATRIC website (Genbank acc number: CP000721, CP002109; Bioproject: PRJNA18165). The genomes of *Clostridium butyricum* 60E.3.1, *Clostridium colicanis* 209318 (*Clostridium sensu stricto*), *Clostridium bolteae* WAL-14578, *C. clostridioforme* CM201.1*, Clostridium clostridioforme* 2149FAA.1, *C. clostridioforme* WAL-7855 (*Clostridium* cluster XIVa) and, *C. innocuum 6_1_30.1* (*Clostridium* cluster XVIII) were selected from the Broad Institute (Genbank Bioprojects: PRJNA64855, PRJNA64873, PRJNA46383, PRJNA64871, PRJNA46389, PRJNA46391, PRJNA39359, respectively).

### Annotation of genome sequences and ortholog groups

Automated genome annotation was generated at the Broad Institute. Additional information was obtained through a proteome analysis pipeline including protein domains search by RPS-Blast with the NCBI Conserved Domain Database [37] and, manual annotation. To improve annotation of the genomes, we determined ortholog groups accross our dataset by using the single-linkage algorithm of BlastClust (NCBI Blast package) at a high stringency level (S90-L0.9). If necessary, a protein sequence was (re)annotated with the ortholog group whose average Blast similarity was highest. In addition, CDSs named according to their position on contigs (ordered locus number) allowed to identify conserved synteny between genomes. Annotation of clusters was also conducted by homology search against the Clusters of Orthologous Groups (COG) protein database [38].

### Detection of putative resistance genes

Antibiotic resistance genes were searched for by using BLASTp at various stringency levels against the Antibiotic Resistance Genes Database (ARDB) on a local machine [39]. Manual sorting of similarities and positive matches of the predicted protein sequences permitted to select manually the closely related AR proteins, and to assign functional annotation.

Putative extrachromosomal elements (e.g., ICEs or Transposons) were detected in the genomes of *C. clostridioforme* and *C. bolteae* by using BLASTp through the ICEberg database [40] upgraded with a personal collection of mobile elements from Firmicutes. A visual inspection of microsynteny (small scale of synteny) was also performed in regions surrounding resistance genes in order to detect mobile elements (e.g. transposase encoding genes) or their traces.

## Additional files

Additional file 1: Table S1. Lactose operons of *C.bolteae* and *C. clostridioforme*. (XLSX 10 kb)
Additional file 2: Table S2. Overview of the three flagellar loci found in *C. bolteae*. (XLSX 14 kb)
Additional file 3: Table S3. Overview of the flagellar locus found in *C. clostridioforme*. (XLSX 13 kb)
Additional file 4: Table S4. Metabolic pathways for butyrate. (XLSX 11 kb)
Additional file 5: Figure S1. Distance based phylogenetic tree of genes coding for butyrate kinase (*buk*). (PNG 721 kb)
Additional file 6: Figure S2. Schematic view of operons coding for glycopeptides antibiotic resistance. Canonical organisation and CDSs present in genomes of *C. bolteae* and *C. clostridioforme* were shown. Percent sequence identity and percent positive substitutions with the nearest homolog of each CDS were indicated. (PNG 409 kb)
Additional file 7: Figure S3. Distribution of genes coding for efflux pumps (potentially involved in antibiotic resistance) in the core genomes of the two species. (PNG 562 kb)
Additional file 8: Figure S4. Distance based phylogenetic tree of genes coding for rifampin resistance (*Arr*). (PNG 995 kb)

## Abbreviations

ARDB: Antibiotic resistance gene database
CDS: Coding DNA Sequence
COG: Clusters of orthologous groups
MIC: Minimum inhibitory concentration
NGS: Next generation sequencing
