# Supplementary figures and images for: Comparative genomics of Clostridium bolteae and Clostridium clostridioforme reveals species-specific genomic properties and numerous putative antibiotic resistance determinants
Source: BMC Genomics. 2016 Oct 21;17:819. doi: 10.1186/s12864-016-3152-x (PMC5073890; doi:10.1186/s12864-016-3152-x)

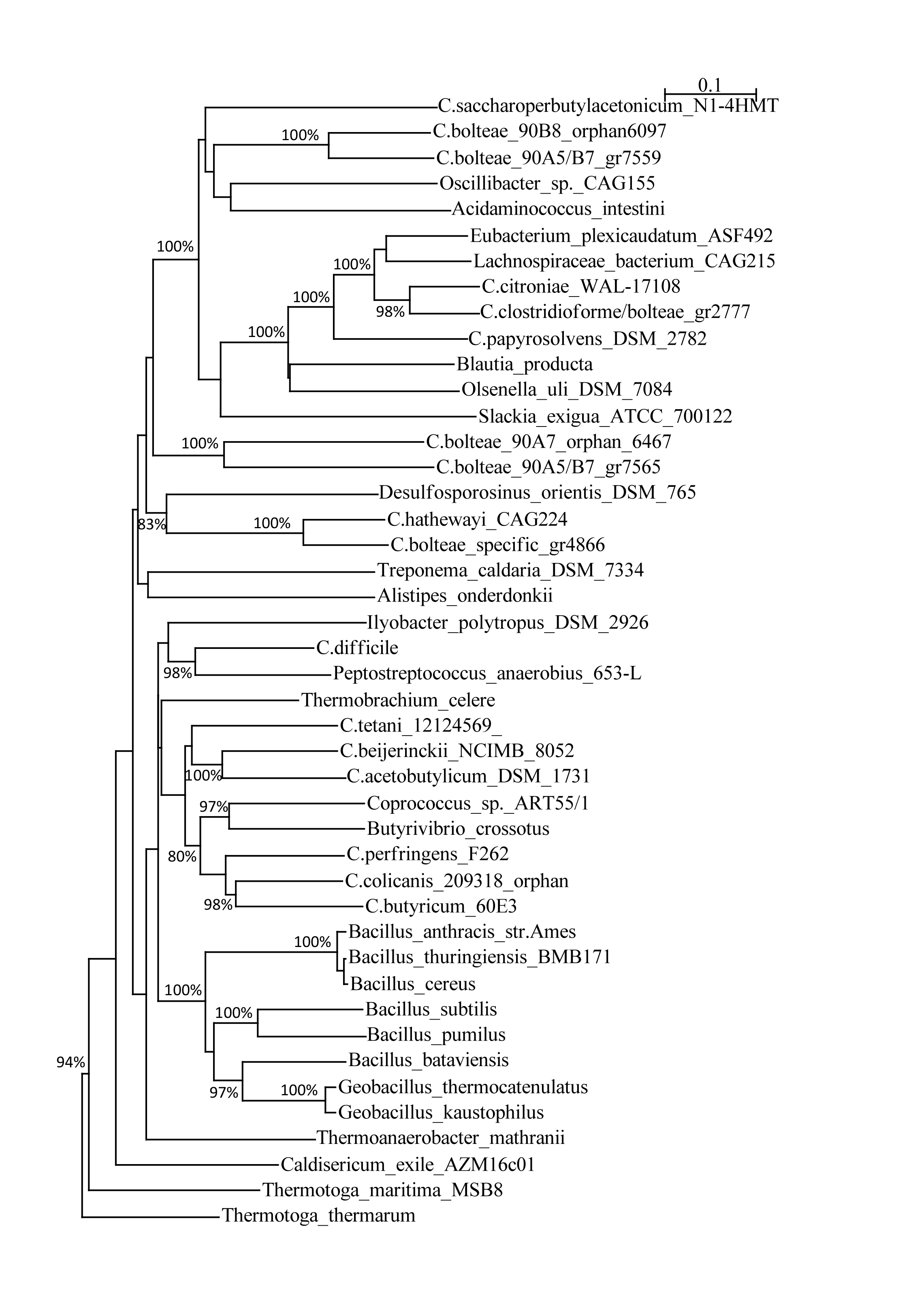

Supplement: Additional file 5: Figure S1. — Distance based phylogenetic tree of genes coding for butyrate kinase (buk). (PNG 721 kb) [file 12864_2016_3152_MOESM5_ESM.png]

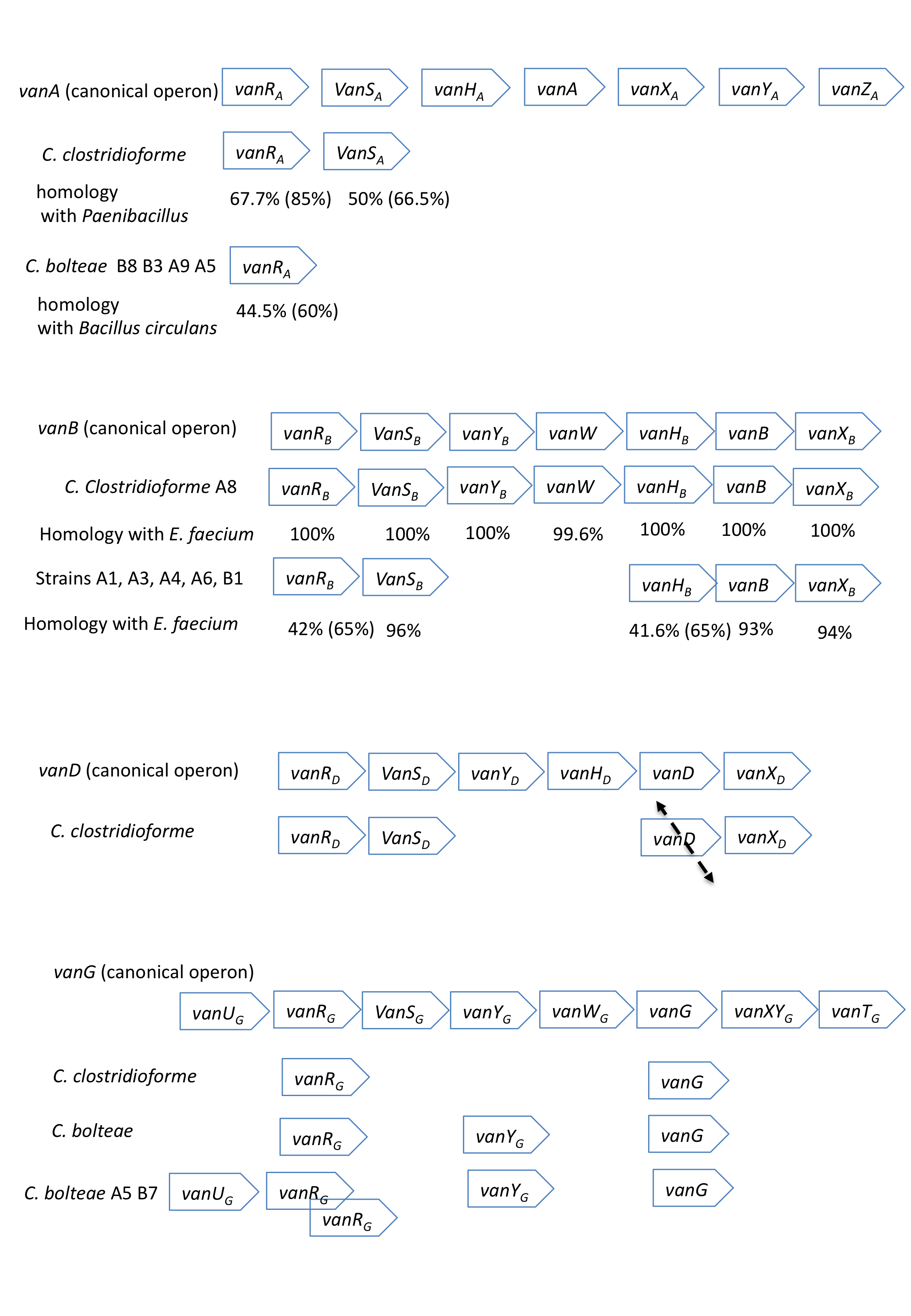

Supplement: Additional file 6: Figure S2. — Schematic view of operons coding for glycopeptides antibiotic resistance. Canonical organisation and CDSs present in genomes of C. bolteae and C. clostridioforme were shown. Percent sequence identity and percent positive substitutions with the nearest homolog of each CDS were indicated. (PNG 409 kb) [file 12864_2016_3152_MOESM6_ESM.png]

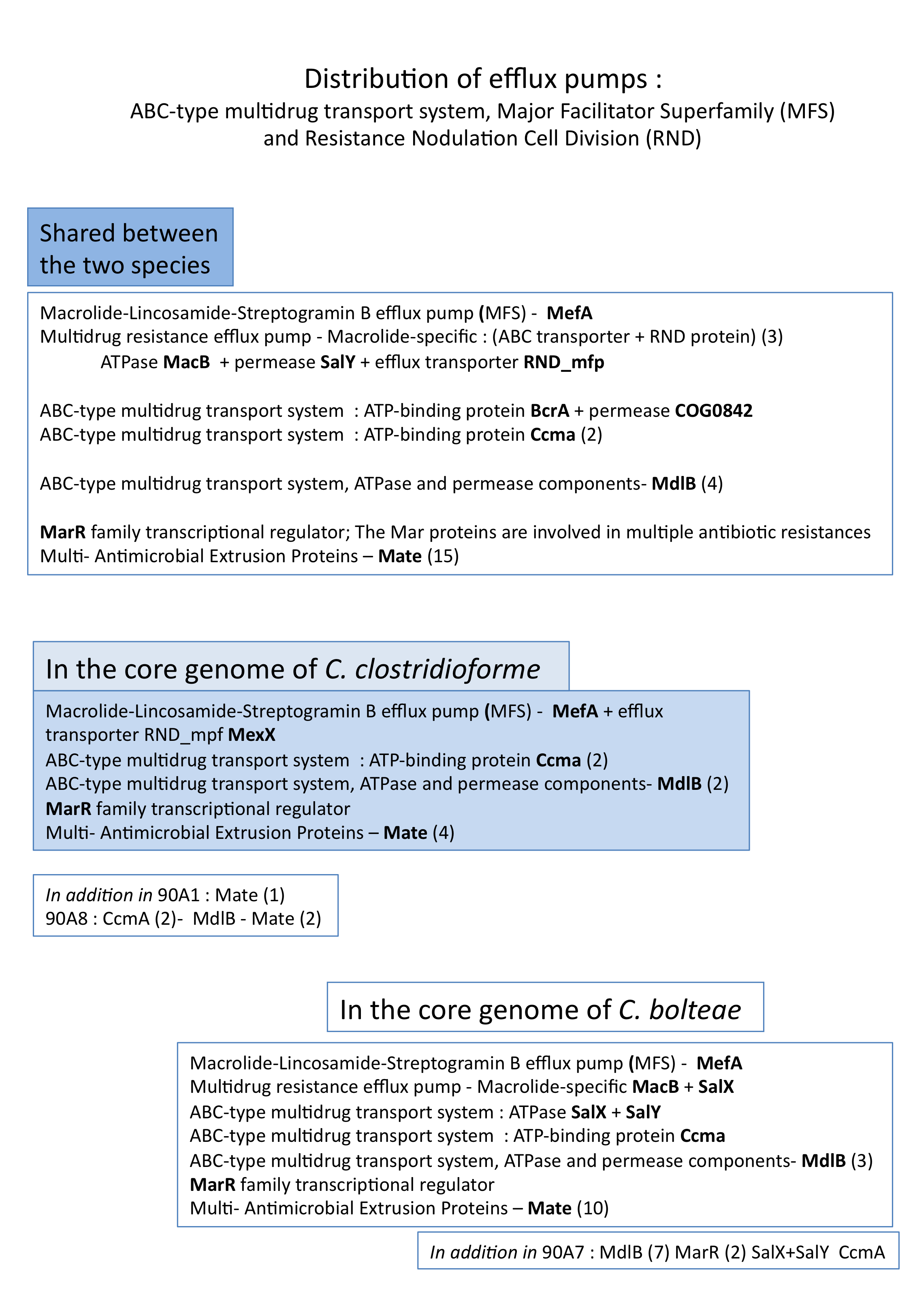

Supplement: Additional file 7: Figure S3. — Distribution of genes coding for efflux pumps (potentially involved in antibiotic resistance) in the core genomes of the two species. (PNG 562 kb) [file 12864_2016_3152_MOESM7_ESM.png]

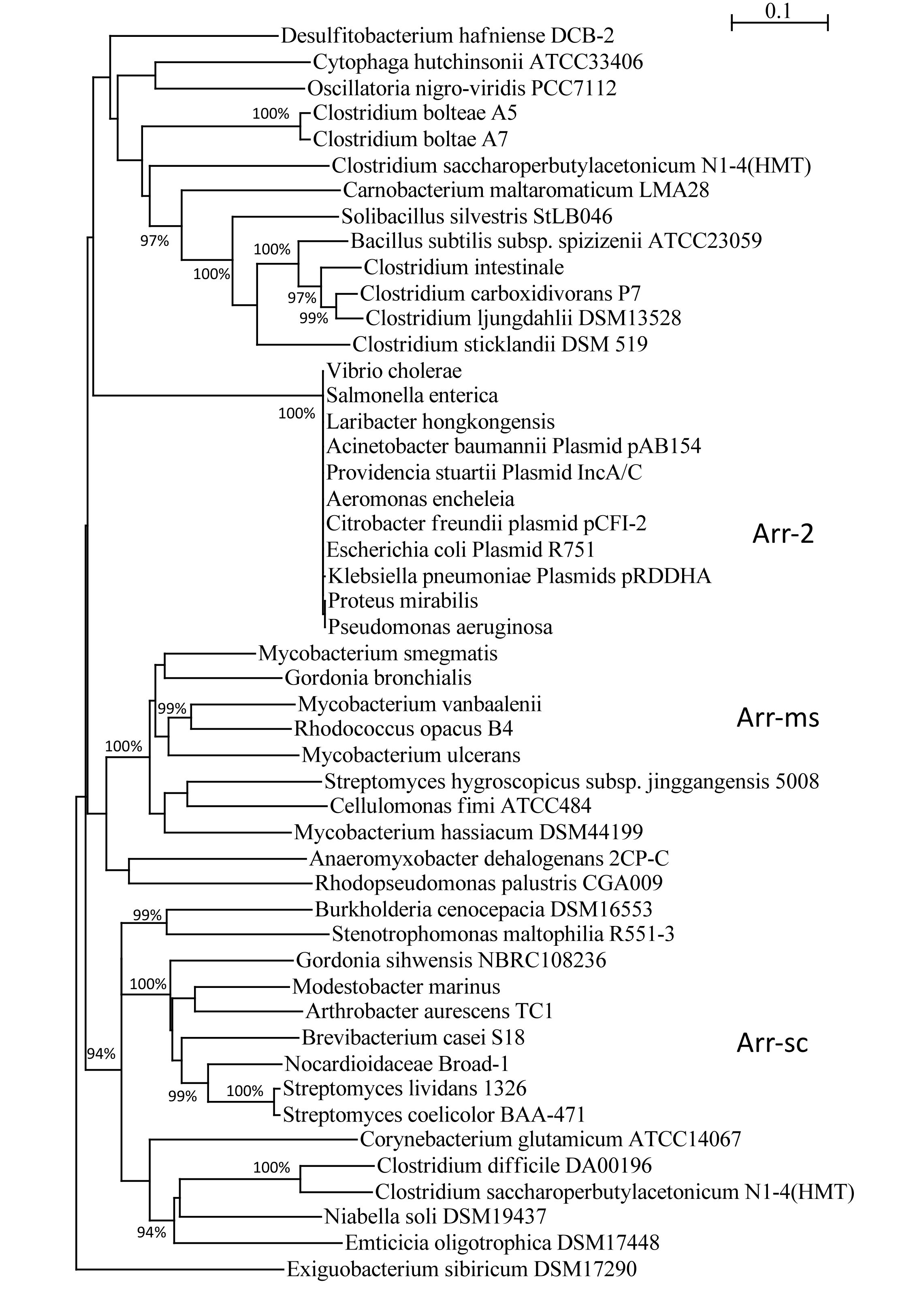

Supplement: Additional file 8: Figure S4. — Distance based phylogenetic tree of genes coding for rifampin resistance (Arr). (PNG 995 kb) [file 12864_2016_3152_MOESM8_ESM.png]
